# Recent Developments in Ion-Sensitive Systems for Pharmaceutical Applications

**DOI:** 10.3390/polym13101641

**Published:** 2021-05-18

**Authors:** Michał Rudko, Tomasz Urbaniak, Witold Musiał

**Affiliations:** Department of Physical Chemistry and Biophysics, Pharmaceutical Faculty, Wroclaw Medical University, Borowska 211, 50-556 Wroclaw, Poland; michalzdzislawrudko@gmail.com (M.R.); tomasz.urbaniak@umed.wroc.pl (T.U.)

**Keywords:** ion-sensitive systems, drug delivery, smart pharmaceutical systems, biocompatible medical devices

## Abstract

Stimuli-responsive carriers of pharmaceutical agents have been extensively researched in recent decades due to the possibility of distinctively precise targeted drug delivery. One of the potentially beneficial strategies is based on the response of the medical device to changes in the ionic environment. Fluctuations in ionic strength and ionic composition associated with pathological processes may provide triggers sufficient to induce an advantageous carrier response. This review is focused on recent developments and novel strategies in the design of ion-responsive drug delivery systems. A variety of structures i.e., polymeric matrices, lipid carriers, nucleoside constructs, and metal-organic frameworks, were included in the scope of the summary. Recently proposed strategies aim to induce different pharmaceutically beneficial effects: localized drug release in the desired manner, mucoadhesive properties, increased residence time, or diagnostic signal emission. The current state of development of ion-sensitive drug delivery systems enabled the marketing of some responsive topical formulations. Concurrently, ongoing research is focused on more selective and complex systems for different administration routes. The potential benefits in therapeutic efficacy and safety associated with the employment of multi-responsive systems will prospectively result in further research and applicable solutions.

## 1. Introduction

The precise delivery of an active pharmaceutical ingredient to the site of action in a controlled manner is one of the primary aims of research conducted in the field of pharmaceutical technology. With an increasing number of pharmacologically active macromolecules, i.a., proteins, the design of an accurate drug delivery system (DDS) capable of effective drug protection gains great attention. Numerous methods were evaluated to provide these beneficial features [1].

One of the extensively researched concepts in the field of drug delivery is focused on stimuli-responsive systems, also termed “smart” carriers. Various chemical and physical stimulants may serve as triggers for drug release. Most of the proposed carriers respond to the changes in temperature and pH value; nonetheless, systems sensitive to the magnetic field, electromagnetic radiation, redox potential, and enzyme presence were reported [2]. In this review, we aim to summarize recent advancements in the development of ion-responsive formulations. Changes in homeostatic ionic strength and composition may be exploited as the trigger, for example, for drug release, phase transition, or diagnostic signal emission. The ions present in physiological fluids and on the surface of mucous membranes are potential stimuli for mucoadhesive and topical formulations. Additionally, a number of medical conditions are accompanied by a change in ionic concentration or may be caused by the presence of exogenic toxic ions present in the environment. Increased Ca^2+^ serum concentrations were linked to various vascular and bone diseases, Zn^2+^ concentrations are considerably higher in nervous tissue, particularly in synaptic vesicles, and changes in Fe^3+^ concentration may indicate anemia and several different diseases [3,4,5]. Furthermore, due to ongoing technological progress, the rise in potentially harmful heavy metal pollution is observed [6]. The development of safe carriers for chelating agents, which are released in response to particular toxic ions, may find an application in industry. Due to the critical role of ion concentrations in different body compartments, the homeostatic mechanisms do not allow significant fluctuations. Therefore, the proposed DDS should be capable of selective response to minor variations in physiological concentration.

**Figure 1:**
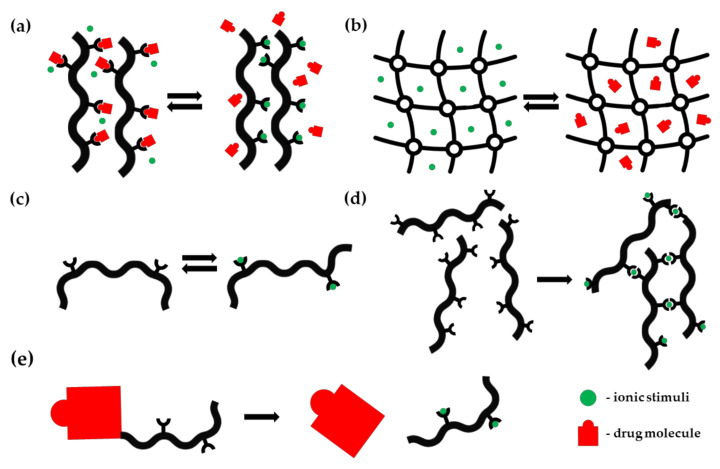
Possible mechanisms of ion-dependent response of pharmaceutical systems: (**a**) ion exchange in polymer resins; (**b**) ion exchange in porous materials; (**c**) conformational change; (**d**) ionic crosslinking; (**e**) prodrug activation.

Various vehicles exhibiting ion sensitivity were proposed, including prodrugs, synthetic polymeric architectures, metal-organic frameworks, polynucleotide structures, or liquid crystals. In this review, we summarize the most recent and distinguished ion-sensitive DDSs divided into two groups, according to their selectivity response toward ions. Within these two main categories, proposed DDSs were classified according to their structure, which is frequently related to the sensitivity mechanism and potential applications (Figure 1).

Among currently available approaches, non-selective topically administered in situ gelling DDSs based on biopolymers are researched most accurately, while more elaborated and selective structures are most often in the initial stage of development. Additionally, multi stimuli-responsive systems aiming at particular microenvironments in the human body gained considerable interest. The possibility of higher therapy efficacy and improved safety offered by an ion-responsive DDS will likely result in further progress and the development of clinically applicable strategies.

## 2. Non-Selective Ion-Sensitive Formulations

There is a considerably large group of formulations that respond to ions generally, without any specific sensitivity towards particular ions or valence. Polymer-based formulations included in this category react to ions in a defined concentration range. Ion-dependent responses include most often gelling and swelling associated with the supramolecular rearrangement of polymeric architectures. The process of ion-dependent gelling is mainly utilized in ocular and nasal formulations. In this part, non-selective ion responsive systems were described and categorized according to the ion-sensitive agent (Table 1).

### 2.1. Gellan Gum

Gellan Gum (GG) is a linear polysaccharide composed of two D-glucose molecules, one L-rhamnose, and one D-glucuronic acid, in a structural unit. In the native form of GG, one acetyl moiety per two repeating units is bound to a glucose molecule. Additionally, there are commercially available deacylated variants described as low acyl GG. The deacylation degree significantly influences the gelation process and mechanical properties of GG gel [7]. A high acetylation degree results in soft gel formation, whereas a low acetylation degree leads to the formation of the stiff gel structure. Due to D-glucuronic acid presence in the structural unit, GG is polyanionic in its deprotonated form. The mechanism of ion-assisted gelling is linked to the formation of cation-induced crosslinking of a double-helical polysaccharide structure, a process sensitive to temperature changes. GG has the ability to form gels after exposition to different metal ions and, in a less pronounced manner, to hydrogen ions. Gels obtained in the presence of divalent ions such as Ca^2+^, Mg^2+^ are less viscous and form at significantly lower concentrations in comparison to monovalent cations, e.g., Na^+^ or K^+^. The minimum gelling concentration of monovalent ions is 100 mM, while for divalent ions, it is equal to 5 mM [7]. The concentrations required for GG gelation in contact with physiological fluids can be found in the mucosal nasal fluid [8], blood [9], and tear film [10]. The experimental nasal formulations are frequently modified by the supplementation with the ion-sensitive GG. The presence of GG prolongs drug residence time in the nasal mucosa, and thus it may enhance the beneficial effects of the intranasal administration route, e.g., avoidance of first-pass effect and decrease in systemic side effects. A combination of modern drug carriers such as nanoparticles with GG may increase the concentration of drug in targeted organs e.g., in the brain. In many cases, formulations utilizing GG as a gelling agent in the nasal cavity proved to be safe and effective. The most often applied concentrations of GG were in the range of 0.3% to 0.5% (*w/v*), whereas the 0.5% (*w/v*) concentration was preferred due to the plausible balance between viscosity, mucoadhesive properties, and impact on drug release profile.

#### 2.1.1. Nasal Formulation

The rapid elimination of poorly soluble drug suspension from the nasal cavity due to mucociliary beating is a significant hindrance for traditional nasal formulations. Application of in situ gelling systems may result in prolonged contact between drug molecules and the vascularized tissues capable of drug absorption. Moreover, low drug diffusion rates, accompanied by a high gel viscosity may result in prolonged drug release. Mometasone, a gel-based carrier designed by Xin-guo Jiang et al., took advantage of these characteristics A nasal DDS based on xanthan gum and 0.5% (*w/v*) GG resulted in a favorable response in animal models compared to the traditional suspension-based system [11]. The nasal drug administration route proved to be beneficial in the alleviation of symptoms linked to motion sickness. Mucoadhesivity is one of the features enabling a bioavailability increase and an overall better therapeutic response in the nasal application. In situ gelling formulation based on 0.3% deacetylated GG and 0.15 carbopol 934 P resulted in a significant mucoadhesive force allowing a prolonged exposition of the dimenhydrinate loaded system to the absorptive tissues. The evaluated formulation showed a slightly negative effect on the nasal epithelium in the animal model [12]. Incorporation of an ion-sensitive gelling agent to the colloidal DDS may prolong residence time. This approach was employed in the design of the curcumin-loaded microemulsion system for nasal drug delivery. Deacetylated GG in the concentration of 0.3% was found to be a suitable ion-sensitive gelling agent for described self-emulsifying system [13]. Nasal drug application is considered a possibly favorable brain tissue delivery path [14]. Therefore, there were attempts to design intranasal systems aiming to increase drug concentration in the brain structures via the prolonged exposure of nasal mucosa to the drug carrier. The formulation including solid lipid nanoparticles with paeonol suspended in 0.4% deacetylated GG as an in situ gelling agent, was employed to alleviate the first-pass effect and to enable brain delivery via the olfactory nerve pathway [15]. Resveratrol loaded nanosuspension, based on 0.6% (*w/v*) deacetylated GG, was employed to exploit an analogous delivery route. The drug concentration in brain tissue increased in the animal model compared to the intravascular administration, and the effect was prolonged. Moreover, the amount of resveratrol distributed in other organs was lower than in brain tissue and was eliminated significantly faster [16]. Employment of 0.5% (*w/v*) GG and 0.15% (*w/v*) xanthan gum enabled brain favoring donepezil delivery. Some advantages were revealed, including high donepezil concentrations in the brain tissue and a significant decrease in drug concentration in the liver and other vital organs compared to a marketed oral formulation [17]. In conclusion, intranasal systems combining in situ gelling properties with contemporary drug carriers may be promising systems for brain drug delivery.

#### 2.1.2. Ocular Formulations

Topical drug formulations are frequently used in the treatment of ocular diseases. The most notable topical ocular drug form are eye drops, with specific local activity and uncomplicated administration. Several hindrances limit the extensive application of eye drops, including the short residence time, resulting in limited absorption and penetration rates of the active compound. Additionally, the eye drops that flow into the lacrimal canaliculus may result in an escalation of side effects. The introduction of in situ gelling agents, i.e., GG, may help to overcome the issues. Some eye-related disorders require prolonged or nearly zero-order drug release. Glaucoma, the incurable disease leading to vision impairment and blindness in the final stage, may be treated with several drugs that can mitigate and even stop disease progression. Most often, they are administered in eye drop form, but due to the short residence time, they require a frequent application to provide sufficient therapeutic concentration. In order to improve eye drop performance, GG was employed in a few strategies. The most straightforward approach to obtain GG-based in situ gelling eye drops was a combination of the drug brinzolamide and GG with a 95% degree of deacetylation. GG in the concentration of 0.5% (*w/v*) was determined as the most beneficial due to both desirable stiffness and optimal residence time of the in situ formed gel. In vitro trials showed that, compared to market eye drops, formulations containing GG release brinzolamide slower and without a burst of release during the first two hours of exposition to artificial tear fluid. Measurements of intraocular pressure in the animal model showed that in situ gelling formulation provided prolonged drug activity without ocular irritation [18]. Gayatri et al. described the complex formulations including GG: carbopol 934 P as a pH-sensitive mucoadhesive agent and benzododecinium bromide as a preservative and corneal penetration enhancer. The experimental formulation was developed via Box-Behnken design and compared to the commercially available TIMOPTIC-XE^®^, which also contained GG as an in situ gelling agent. Compared to the marketed product, the optimized formulation had a comparable release profile in vitro with less pronounced concentration fluctuations. The same observations were made for the measurements of intraocular pressure in animal models; the formulation was stable and well-tolerated [19]. Another approach is based on liposomes as drug carriers combined with deacetylated GG, in order to address low active pharmaceutical agent bioavailability. The deacetylated GG concentration of 0.4% (*w/v*) was found to be optimal due to the most beneficial viscosity and release rate. Supplementation with deacetylated GG resulted in a less pronounced burst release and an overall decrease in drug release rate in comparison to an aqueous suspension of liposomal timolol maleate. Measurement of residence time via fluorescence imagining proved that in situ gelling liposomal timolol eye drops exhibited the longest contact time with eye surface. A comparison of the intraocular pressure after exposition to the evaluated system and reference timolol eye drops showed a more pronounced response in the case of the in situ gelling formulation [20]. In ocular infections, topical DDSs can simultaneously decrease systemic impact and locally increase therapy efficiency. The potency of some antibiotics depends on the time of their presence in a site of action at the proper concentration. Therefore, beneficial in situ gelling systems are employed in experimental antimicrobial formulations for ocular administration. Asgar Ali et al. utilized pefloxacin as an antibiotic agent combined with a GG solution. An increased concentration in GG resulted in a slower release rate and a more linear drug liberation. As a result, GG also influenced the antimicrobial properties of the formulation. In vitro tests conducted via the cup plate method showed that optimized formulation had a bigger area of inhibition than marketed pefloxacin eye drops [21]. An analogous approach was employed in the GG-based ocular system loaded with moxifloxacin and ketorolac as an anti-inflammatory agent. Dissolution tests of formulations employing GG in concentration range 0.1–0.25% (*w/v*) confirmed the influence of ion-sensitive polysaccharides on the release of both drugs [22]. A combination of liposomal drug carriers with an in situ gelling ion-sensitive matrix was proposed as an efficient delivery approach for the lipophilic antifungal drug—natamycin. A comparison with the marketed drug suspension confirmed the advantages of the evaluated system, a superior corneal permeability, as well as prolonged residence time [23]. Due to the distinctive anatomical structure of the human eye, systemic delivery of lipophilic pharmaceutically active agents of high molecular weight is extremely difficult. Therefore, local administration is often employed as a strategy to achieve high intraocular concentration. An example of such an approach was described by Chetoni et al. Application of cyclosporine-A loaded micelles entrapped in ion-sensitive in situ gelling matrix resulted in improved drug solubility and enhanced residence time in tear fluid [24]. A distinct example of a dry ocular DDS composed of GG-pullulan electrospun nanofibers was proposed. Immediate gel formation after lens application in the animal model was achieved. Model drug residence time on the ocular surface was significantly longer compared to a drug applied in the form of eye drops [25].

#### 2.1.3. Other Applications

Prior discussed approaches demonstrated the two most commonly described examples of GG’s use in pharmaceutical applications—ocular and intranasal. However, there are reports of this in situ gelling agent employment in other ion-rich environments. Wound dressings are one of the medical supplies which may potentially benefit from GG employment. Carboxymethyl chitosan-based wound dressing enriched with GG-derived microparticles exhibited prolonged release of the antibacterial drugs—tetracycline and silver sulfadiazine [26]. Another study focused on burn wounds treated with a collagen-GG crosslinked network, employed as a carrier for cells promoting regeneration. The obtained interpenetrating network improved early wound closure, reduced inflammation, and promoted regeneration for third-degree burn wounds [27]. GG is also utilized in formulations for hard tissue regeneration enhancement. P. Matricardi et al. developed an ion-responsive formulation for bone and cartilage defects treatment. The described system consisted of two solutions, which, after contact, formed a bioadhesive hydrogel via ion-activation. The primary solution consisted of hyaluronic acid and CaCl2, whereas the secondary solution was an aqueous GG solution. Hyaluronic acid with Ca^2+^ was applied to the defect cavity, followed by dropwise application of the GG solution. Initially, a two-layered environment inside the cavity was formed; subsequently, diffusion of the formulation components promoted further hydrogel formation. The hydrogel provided a good environment for osteoblasts proliferation; the formulation had plausible adhesiveness and durability [28]. A clotrimazole-loaded formulation for anti-fungal dental application was obtained via a combination of GG as a gelling agent and Ca^2+^ ions in the form of a citrate complex. In the oral cavity, Ca^2+^ was released from the citrate complex due to a slightly acidic environment. Thus, the Ca^2+^ level sufficient for gelation was obtained. The employment of GG enhanced the residence time of clotrimazole on the surface of the oral cavity improving the anti-fungal activity of the formulation [29]. Medical procedures requiring intrauterine device insertion are often avoided due to associated significant pain. Employment of local anesthetics can mitigate pain during intrauterine procedures. An intravaginal formulation composed of GG as an ion-responsive agent was proposed as a carrier of lidocaine for local analgesia. The investigated system exhibited good biocompatibility in the animal model. The formulation was employed during different intravaginal medical procedures performed on women volunteers and provided significant pain alleviation [30].

### 2.2. Alginates

Alginates are polysaccharides occurring naturally in brown algae and soil bacteria. The backbone of the alginate chain consists of α-L-guluronic acid and β-D-mannuronic acid residues linked with 1–4 bonds. The arrangement of moieties present in the alginate molecule is variable and depends on the source organism or even tissue of origin. The molecule of alginate consists mainly of randomly distributed guluronic and mannuronic monomers, with locally ordered fragments. Both arrangement and monomer ratio strongly influence polymer chain stiffness and results in varied physical and mechanical properties. Guluronic acid units, especially if present in homopolymeric sequences, are capable of interacting with multivalent cations, notably Ca^2+^. As a result, alginates are capable of ion-dependent gel formation via electrostatic crosslinking. Therefore, there is an applicative potential for the alginate-based in situ gelling system in divalent cation-rich environments, e.g., in the ocular surface, nasal cavity, or in the vascular bed. Moreover, alginates exhibit temperature-independent gelation, which allows thermal processing of obtained ion-sensitive formulations [31].

#### 2.2.1. Ocular Formulations

Alginates are employed in the preparation of in situ gelling ocular formulations based on ion-sensitive behavior, similar to GG. Ion-induced alginate gelation is one of the features that can address the issue of low drug bioavailability and short drug residence time. Significant attention in the field of ocular infections was paid to antimicrobial drugs from the group of quinolones. The use of quinolone-loaded sodium alginate as an ion-sensitive in situ gelling agent combined with cellulose derivatives was reported. Sodium alginate containing 40% of guluronic and 60% mannuronic acid, combined with HPMC as a viscosity-enhancing agent, formed an ion-responsive formulation. A higher alginate concentration resulted in the increase in formulation gelling capacity; the same effect was observed in systems containing a constant alginate concentration and increasing content of 90 kDa HPMC. A profile of gatifloxacin release from the optimized formulation was characterized by a less pronounced burst release effect and provided sustained drug release for 8 h. The formulation was well tolerated in the animal model; no ocular damage and iatrogenic abnormalities were observed [32]. An analogous ion-sensitive system based on sodium alginate and HPC was developed as a potential ofloxacin carrier. An optimized formulation containing 1.5% (*w/v*) alginate and 0.5% (*w/v*) HPC exhibited, similarly to the abovementioned, an 8 h sustained release of the drug [33]. Alginate-based ocular formulation designed to carry the lipophilic anti-inflammatory drug nepafenac was investigated [34]. The active pharmaceutical ingredient was complexed with hydroxypropyl-β-cyclodextrin to improve water solubility and incorporated into alginate and an HPMC solution. An optimized formulation containing 0.3% (*w/v*) alginate was compared to the marketed Nevanac^®^, an ophthalmic suspension of nepafenac. The concentration of nepafenac in the cornea was significantly higher in the case of the investigated formulation compared to reference suspension.

#### 2.2.2. Other Applications

A sodium alginate-based oral in situ gelling DDS loaded with paracetamol capable of forming a gel in the stomach was reported [35]. The administered solution contained Ca^2+^ ions complexed in citrate form to ensure gelation in the acidic stomach fluid, as a result of Ca^2+^ release via H^+^ substitution. The formed gel provided paracetamol release for 6 h and a release profile close to the reference suspension form. An alginate-based system for prolonged rifampicin release was proposed for administration in the form of poly (lactic-co-glycolic acid) microspheres suspended in sodium alginate solution. The described formulation was administered by endotracheal intubation in the animal model. The employment of alginate resulted in a significant delay in drug delivery and 24 h adhesion. Presented results indicate that the described formulation could be useful for the interventional treatment of tuberculosis [36]. A two-component alginate injectable formulation capable of in vivo matrix formation was reported. Alginate was employed as a gelling agent alongside a Ca^2+^ reservoir in the form of microspheres for inducing in vivo gelation. Microspheres in an alginate solution may be applied as carriers for pharmacologically active substances e. g., interleukins. After formation, gels were infiltrated by host cells. The authors speculate that such a formulation could be employed for cell incorporation [37].

### 2.3. Other Carriers

DDSs of another origin were also utilized in order to obtain non-selective ion-sensitive products potentially applicable in various drug delivery strategies. A complex ion-sensitive DDS for ocular application in the form of a contact lens was evaluated. The main barrier in drug delivery via contact lenses is drug leak during storage. This disadvantage can be addressed by the application of ion-sensitive macromolecular architectures. The described lenses were made out of a silicone outer layer and a poly(styrene-divinylbenzene) sulfonic acid resin. The resin was dispersed in a copolymeric matrix and served as a betaxolol reservoir. A prepared DDS, stored in distilled water, due to a lack of ions capable of drug substitution, did not release the drug during a prolonged period. However, after exposition to the artificial tear fluid, the drug was liberated with an observable burst release in the first 4 h and a subsequent slower discharge in the following 6-days [38]. Previously discussed ion-sensitive systems were designed mainly for topical applications. Nevertheless, some ion-dependent formulations may be beneficial in parenteral administration. Y. Xu et al. described ion-sensitive microparticles targeting lung cancer tissue [39]. The particles were electrostatically loaded with interferon α-2b. Nanoporous microspheres were obtained using carboxymethyl chitosan and subsequently loaded via electrostatic interaction with the chemotherapeutic agent. Due to the ionic nature of the interaction between the drug and the carrier, the protein release occurred via an ion-exchange mechanism. Physiologically occurring cations substituted positively charged interferon and resulted protein release from the carrier in the target tissue. The obtained formulation provided a sustained-release and drug accumulation in the lungs. The electrostatic protein binding was investigated with micro-sized gel particles of carboxymethyl cellulose crosslinked with sodium trimetaphosphate [40]. Carriers prepared with various crosslinker concentrations were loaded with lysozyme as a model protein. The impact of ionic strength on the protein adsorption and swelling degree was investigated. An increase in ionic strength resulted in a decline in protein uptake and a reduced swelling degree. Authors suggested that the decrease in lysozyme uptake in the presence of a higher ion concentration was associated with the salt screening phenomenon resulting in reduced attraction between charged microgel and lysozyme molecules. The same mechanism was probably responsible for the mitigation of repulsion between polymer chains and subsequent decrease in swelling degree. The ion responsive properties of dextran-poly(acrylic acid) copolymer were evaluated in ibuprofen-loaded particles. It was observed in the release experiments that ion presence in the acceptor medium significantly decreased ibuprofen release rate, especially in an acidic environment.

Furthermore, an increase in ionic strength resulted in increased particle hydrodynamic diameters. According to the authors, ion introduction had a significant impact on hydrogen bonding, ionic interactions, and surface charge, which subsequently affected particle size and release rate of ibuprofen [41]. Eudragit RS/LS coated beads were examined to elucidate the mechanism of diltiazem release. The ionic strength of the release media in a particular range resulted in higher release rates and a shorter drug release lag time. This observation was linked to the mechanism based on the exchange of a counterion of quaternary ammonium groups present in the eudragit molecule. The initial increase in ionic strength promoted a faster exchange of chloride anions and thus a faster release and shorter lag time to a certain ionic strength value. A further increase in ionic strength had the reverse effect, explained as a result of increased osmotic pressure [42]. Multiresponsive cryogels based on methacrylates and acrylamide-derived polycations exhibited some ionic strength-dependent behavior. The interaction between the counterion and the charged polymer groups led to a decrease in electrostatic repulsion inside supramolecular gel structure and thus a reduced distance between neighboring polymer chains. This phenomenon was manifested as a reduced equilibrium water uptake and may potentially have an impact on the release of the incorporated pharmaceutically active ingredient [43]. A therapeutic system employing porous polyvinylidene fluoride membrane grafted with acrylic acid was proposed as an ionic strength sensitive barrier for drug delivery. Membrane containers filled with solid drug doses were investigated as potential DDSs for propranolol, caffeine, and sodium salicylate. Release experiments in pH 7.0 and varying salt concentration resulted in a diminished release rate of caffeine and propranolol in higher ionic strength. On the contrary, sodium salicylate showed a slight change in release profile when the media ionic strength was varied. It was suggested that ion interaction with polyacrylic chains might result in the rearrangement of polymeric architecture and thus affect membrane permeability for certain molecules [44]. Structurally differing systems based on metal-organic frameworks (MOFs) were described as potential ion responsive carriers. A single crystalline material based on zinc, adenine, and biphenyl dicarboxylic acid was described by Rosi et al. [45]. The pores present in the obtained structures enabled the incorporation of procainamide in its cationic form. A significant ion-dependence during drug release was observed in dissolution studies. The release profile observed in phosphate-buffered saline differed considerably from the profile obtained in the corresponding experiment performed with deionized water. According to the authors, the cation-dependent drug release mechanism was related to the ion substitution phenomenon.

### 2.4. Selective Ion-Sensitive Systems

Except for non-selective formulations, systems responding selectively to certain ions or valence in a particular concentration range may be distinguished (Table 2). Due to distinctive changes in ion concentration accompanying various pathological conditions, such behavior may be extremely beneficial in terms of therapy efficacy and safety.

### 2.5. MOFs

MOFs are structures composed of metal-oxo clusters connected by organic ligands characterized by high porosity. Due to a high pore volume and surface area, they may serve as potential carriers for a variety of molecules, including drugs.

Nanoporous UiO-66-NH_2_ MOF was conjugated with quaternary ammonium salts to obtain a DDS sensitive to the presence of Zn^2+^ ions. Carboxylato-pillar [5] arene caps, with an electron-rich cavity, complexed positively charged quaternary ammonium groups on the MOF surface via a host-guest interaction. This capping mechanism prevented 5-fluorouracil (5-FU) release from the pores. 5-FU liberation occurred after the detachment of the capping agent, competitively complexed by introduced Zn^2+^ ions and the subsequent exposition of the incorporated cargo to the release media. The release rate of 5-FU was determined in different concentrations of Zn^2+^; the increase in release rate was observed in higher Zn^2+^ concentrations. In physiological concentration of Zn^2+^, only 5% of the incorporated dose was released, indicating a possibly low premature drug release after administration. In vitro cytotoxicity of the non-loaded system was negligible in low concentrations [46]. Another Zn^2+^ sensitive system was based on two topological types of indium MOFs. Carrier nanopores served as a 5-FU reservoir, and in the presence of Zn^2+^, the drug was released in an ion-exchange mechanism. The release of different Zn^2+^ concentrations was evaluated, in the lack of Zn^2+^ ions, the release curve of 5-FU achieved a plateau on the level of 50–55% released drug. An increase in Zn^2+^ concentration resulted in higher plateau levels up to 90% [47]. Ion-responsive MOF-based DDSs may also serve as a diagnostic tool. One of the examples of theranostic carriers was obtained using a zirconium-based MOF deposited on the surface of Fe_3_O_4_ nanoparticles. Drug-loaded MOF was modified with 1-(6-bromohexyl) pyridine, which allowed the host-guest capping of MOF nanopores with carboxylate-pillar [6] arenes. The competitive complexing of capping agent with Zn^2+^ and Ca^2+^ in surrounding media resulted in complex detachment, and subsequent exposition of the drug-loaded pores. 5-FU release in media without Zn^2+^ or Ca^2+^ achieved a plateau below the level of 15% of the incorporated drug after 2 h. Approximately a two-fold plateau level was observed in the presence of divalent ions [48].

### 2.6. Liposomes

A few approaches were employed in order to modify liposomal bilayer structure with ion-responsive molecules to provide ion sensitivity. Michael D. Best et al. reported a Ca^2+^ activated release from the liposomal system obtained via the introduction of an ionic switch anchored in the phospholipid bilayer via lipophilic hydrocarbon chains. The tetracarboxylate chelating site of the sensor undergoes a conformational change after Ca^2+^ complexing, which results in liposome membrane disruption and encapsulated drug release. The triggering effect of Ca^2+^ was pronounced, whereas the presence of K^+,^ Na^+^, and Zn^2+^ had no impact on release. Furthermore, a significant change in carrier hydrodynamic diameter was observed [49]. Ion responsive liposomes employed as theranostic systems, selective Hg^2+^ sensitive carriers for detection and neutralization of Hg^2+^ was reported. Liposomes with pegylated phosphatidylethanolamine embedded in their bilayer were loaded with fluorescein as the indicator and meso-2,3-dimercaptosuccinic acid as the chelating agent. The mechanism of Hg^2+^ mediated release is based on the interaction between Hg^2+^ and phosphatidylethanolamine headgroups, which results in molecule reorientation and destabilization of the liposomal membrane. Subsequently, indicator release occurs, and concurrently discharged chelating agent neutralizes Hg^2+^. Further investigation confirmed high selectivity towards Hg^2+^ in comparison to other metal cations. An in vitro cell viability evaluation confirmed that the presence of liposomes containing a chelating agent increased cell viability in the presence of Hg^2+^ [50]. The Cu^2+^ responsive liposomal system was obtained by employing egg lecithin to form a carrier membrane enriched with an ion-sensitive switch. Employed Cu^2+^ sensitive agents were derivatives of 3,7-diazabicyclo[3.3.1]nonan-9-one obtained by substituting secondary amine groups with alkyl chains, which enabled incorporation in carrier membrane. Conformational changes induced by Cu^2+^ in incorporated switches led to the formation of membrane defects and a subsequent leak of liposome cargo. Fluorescent dye incorporated in obtained liposomes was released exclusively in the presence of Cu^2+^ [51].

### 2.7. Other Carriers

The in situ gelling ion-responsive peptide-based system that could be potentially employed in prostate cancer therapy was proposed. A novel D3F3 forky peptide was synthesized and employed as a Zn^2+^ responsive agent for in situ hydrogel formation in the targeted organ. The determined gelation-inducing Zn^2+^ concentration of D3F3 was lower than observed physiological levels. Exposition to other ions present in biological fluids in physiological concentrations did not trigger gel formation. The in vitro model showed that the peptide component of the system exhibited no toxicity, whereas the one loaded with doxorubicin had a higher efficacy than doxorubicin alone [52]. The mesoporous nanoparticle system was designed to respond selectively to Pb^2+^. The carrier was based on epoxidated silica particles loaded with fluorescein, which was released specifically in the presence of Pb^2+^. Silica particles were surface modified with a substrate DNA strand, which was subsequently hybridized with a biotinylated Pb^2+^ specific DNAzyme. The obtained pore “gate” was capped with avidin via the biotin component of the DNAzyme. In the presence of Pb^2+^, the substrate underwent degradation as a result of enzyme activation and following pore uncapping occurred. The Pb^2+^ triggered fluorescein release, which was proportional to the ion concentration. The impact of other ions on the investigated system was negligible; thus, it was concluded that the system is Pb^2+^ selective [53]. The Fe^2+^ sensitive prodrug designed for Plasmodium infection treatment was developed based on microbial catabolism responsible for the formation of ferrous ions in a host organism. The drug molecules were conjugated with a 1,2,4-trioxolane ring serving as a sensing moiety. In the presence of Fe^2+^, the 1,2,4-trioxolane ring underwent degradation accompanied by iron oxidation. Subsequently, the drug-sensor linker was eliminated, resulting in the activation of a drug molecule. The prodrug was characterized by a superior efficacy in the animal model, compared to a non-conjugated pharmaceutically active molecule [54]. A complex K^+^ and pH sensing system dedicated to drug liberation in a cancer microenvironment based on framework nucleic acid was evaluated by T. Li et al. The system was composed of tetrahedral DNA nanostructures combined with cholesterol molecules, as cell membrane anchoring agent, fluorophores as indicators, and a pharmacologically active AS1411 aptamer. In proper pH and in the presence of K^+^, the system was subjected to supramolecular rearrangement, resulting in aptamer folding and subsequent aptamer release in the form of a G-quadruplex. Simultaneously, the spatial interaction between fluorescent probes became possible, resulting in a change in observed fluorescence [55]. The Ca^2+^ sensitive structure, based on a chiral imprinted cholesteric liquid crystalline polymer, was proposed as a possible system capable of ion serum level monitoring. The thin layer of the liquid crystalline phase was obtained via surface photopolymerization. Due to the presence of benzoic acid derivatives exhibiting an affinity to Ca^2+^, the obtained system manifested a noticeable color change, selectively in the presence of these ions. System response was most pronounced in the concentration range observed in human plasma, and thus it was concluded that the obtained ion-sensitive sensor could be employed as a convenient test for serum calcium levels [56]. The phenomenon of a divalent ion-dependent change in emission spectra was reported for systems assembled via noncovalent interaction between single-strain 30-nucleotide DNA and carbon nanotubes sidewalls. The most pronounced impact on emission energy was observed in the presence of Hg^2+^, which promoted a DNA transition from form B to conformation Z and a subsequent shift in band-gap fluorescence. The observed phenomenon was found to be fully reversible after ion removal. The potential application of heavy metal ion detection in body fluids was suggested. The most pronounced shift was observed in the presence of Hg^2+^; nevertheless, the system exhibited minor affinity towards other ions, which may reduce detection sensitivity [57]. A nasal formulation supplemented with low methoxyl pectin, as an ion-responsive gelling agent, exhibited enhanced adhesiveness. Divalent ions present on the mucosal surface crosslinked the adjacent pectin chains via carboxyl groups resulting in an ordered network. As a result, dripping and throat flow were reduced. The described approach may be beneficial in the nasal administration route due to the prolonged exposure time and irritation alleviation [58]. A novel system composed of two hydrogel elements was proposed. The first gel exhibited an ion-induced adhesiveness towards the second one serving as a binding hydrogel component. The ion-responsive hydrogel was obtained via the grafting of β-cyclodextrin and 2,2′-bipyridyl moieties into polyacrylamide chains. The second binding hydrogel component was prepared by incorporation of N-tert-butyl acrylamide moieties capable of host-guest interaction with β-cyclodextrin. In the native state, 2,2′-bipyridyl moieties are complexed by β-cyclodextrin, and consequently, the adhesive properties were hindered. 2,2′-bipyridyl moieties competitively complexed cations, resulting in the liberation of β-cyclodextrins. The non-complexed cyclodextrins interacted with the N-tert-butyl acrylamide moieties of the binding hydrogel, and adhesion between the two components was observed. The type of cation impacted ion-responsive hydrogel properties leading to various results [59]. A complex system capable of mimicking biological Ca^2+^ was reported. To construct this nanostructure, a copolymer consisting of three components: N-isopropyl acrylamide, acrylamide-[4-(trifluoromethyl)phenyl]-2-thiourea, and DDDEEKC was immobilized on the surface of the nanoporous membrane. DDDEEKC is a Ca^2+^ responsive selective ion-binding heptapeptide. Acrylamide-[4-(trifluoromethyl)phenyl]-2-thiourea is bound to the DDDEEKC peptide via hydrogen bonds formed via thiourea moieties. In a sufficient concentration of Ca^2+^, the hydrogen bonds underwent rearrangement leading to the transition of the copolymer from the globular to the coil form and thus leading to channel blocking. The system was described as highly selective and capable of operating in physiological ion concentrations [60]. A distinctive micellar system releasing cargo in reduced pH in the presence of chloride ions was designed to deliver a platinum-derived antitumor agent. Nanocarriers entered cancer cells via endocytosis, where (1,2-diaminocyclohexane) platinum aqua complexes were activated in the reduced pH and in the presence of nucleophilic Cl^−^ ions. The carrier exhibited enhanced antitumor activity and, due to drug delivery to the tumor cell cytoplasm, avoided drug-resistance mechanisms [61].

## 3. Summary

Ion-sensitive DDSs, with their capability to provide a variety of effects in a controlled manner, are a promising category of medical devices. The mechanisms of carrier response may be based on an ion-exchange phenomenon, non-covalent crosslinking, a salt screening effect, selective complexing, or enzyme activation. This variety of ion-induced phenomena translates into a number of pharmaceutical and pharmacological effects occurring after exposition to these stimuli. The most explored type of ion-responsive behavior is an ion-triggered release of small pharmaceutically active molecules and macromolecular species. Drugs are liberated from the described systems with different selectivity towards particular ions. Non-selective DDSs release cargo mainly via ion exchange and salt screening effect. A release in response to specific ions was achieved in porous MOFs, where pore size and charge were suited for particular ions. Other selective approaches were based on ion-specific molecular switches and macromolecules such as enzymes and polynucleotides. Conformational changes occurring in the presence of specific ions are responsible for the release-inducing phenomena. Apart from drug delivery purposes, the described approaches may be applicable in a DDS suspended in aqueous media to avoid drug leak during storage. In situ gelling matrices enabled increased residence time with an associated change in drug delivery rate. Most of these approaches were based on ionically-crosslinked polysaccharides for topical administration of antimicrobial and antiglaucoma drugs. Nevertheless, DDSs aimed at oral, intrauterine, dental, or intravenous administration were proposed. Polysaccharide-based DDSs were generally well tolerated and non-toxic, which makes them promising candidates for clinical applications. The ion-induced rearrangement of carrier structure also resulted in the emission of diagnostic signals, which in combination with drug release, may be highly beneficial in terms of controlled drug deposition. Especially in the case of chemotherapeutics with a narrow therapeutic index, such an approach may greatly improve treatment safety. Notwithstanding, significantly less attention was paid to the ion-sensitivity approach in comparison to pH-sensitivity, enhanced permeability effect, or thermosensitive DDS [62]. Most of the carriers described above operate in physiological millimolar ion concentrations. However, physiological fluctuations observed in extracellular fluids occurred in a narrow range and varied between individuals, which presumably is insufficient to induce a proper response [63]. On the contrary, more significant variations in ion concentration were observed in the intracellular environment of particular tissues, both in physiological and pathological conditions [64,65,66]. An approach combining active targeting and intracellular ion-dependent drug activation is considered advantageous and was employed in the design of a carrier of drug currently under clinical trials [60]. Due to the variety of available ion-sensitive switches, chelating agents, and ionic polymers, further advancements in the described category of DDSs are expected. In the case of topical formulations, a simple addition of an in situ gelling agent may be an extremely beneficial and safe way to increase therapeutic efficacy. Systemically administered carriers dedicated to more precise drug delivery may benefit from ion sensitivity, mainly by a reduction in premature drug release. However, the most beneficial outcomes are expected as a result of the combination of ion-sensitive properties with other smart drug delivery strategies. The ongoing development of multi-stimuli responsive smart carriers will presumably result in applicable solutions with an ion-sensitive component.

## Figures and Tables

**Table 1:** Summary of non-selective ion-responsive systems for pharmaceutical applications.

Ion-Sensitive Component	Incorporated Substance	Application	Ion-Induced Response	Ref.
Gellan gum	Momentasone	Allergic rhinitis	Prolonged residence in nasal cavity	[11]
	Dimenhydrate	Motion sickness	Alternative administration route	[12]
	Curcumin	n/a	Nose-to-brain delivery	[13]
	Paeonol	Neuroprotection	Nose-to-brain delivery	[15]
	Resveratrol	Neurodegenerative diseases	Enhanced pharmacokinetic profile	[16]
	Donepezil	Alzheimer’s disease	Alternative route of administration	[17]
	Brinzolamide	Glaucoma	Enhanced pharmacokinetic profile	[18]
Gellan gum	Momentasone	Glaucoma	Drug release control	[19]
	Dimenhydrate	Glaucoma	Prolonged residence time	[20]
	Curcumin	Bacterial infection	Enhanced antibacterial activity	[21]
	Paeonol	Bacterial infection	Enhanced pharmacokinetic profile	[22]
	Resveratrol	Fungal infection	Permeability and residence time enhance	[23]
	Cyclosporine-A	Dry eye disease, choroid inflammation	Enhanced solubility and residence time	[24]
	Tetracycline, Silver sulfadiazine	Wound dressing	Sustained drug release	[25]
	Collagen	Wound dressing	Wound regeneration improvement	[26]
	Hyaluronic acid	Bone and cartilage regeneration	Increase in proliferation of osteoblasts	[28]
	Clotrimazole	Dental fungal infection	Prolonged residence on mucous membrane	[29]
	Lidocaine	Local analgesia	Pain alleviation during medical intervention	[30]
Alginates	Gatifloxacin	Bacterial infection	Sustained drug release	[32]
	Ofloxacin	Bacterial infection	Sustained drug release	[33]
	Nepafenac	Anti-inflammatory	Enhanced permeability	[34]
	Paracetamol	Pain and fever therapy	Prolonged release	[35]
	Rifampicin	Tuberculosis infection	Delayed release	[36]
	IL-2	Immunomodulation	Formation of matrix for cell colonization	[37]
Poly (styrene-divinyl benzene) sulfonic acid	Betaxolol	Glaucoma or ocular hypertension treatment	Ion-dependent release	[38]
Carboxymethyl chitosan	Interferon α-2b	Antitumor	Sustained release and lung accumulation	[39]
Carboxymethyl cellulose	Lysozyme	n/a	Gel swelling and protein uptake	[40]
Dextran-poly (acrylic acid) copolymer	Ibuprofen	n/a	Controlled drug release and gel swelling	[41]
Eudragit RS/LS	Diltiazem	n/a	Controlled drug release	[42]
Methacrylate	n/a	n/a	Gel swelling, water uptake	[43]
Acrylic acid grafted polyvinylidene fluoride	Propranolol, caffeine, sodium salicylate	n/a	Controlled drug release	[44]
MOF	Procainamide	n/a	Controlled drug release	[45]

**Table 2:** Summary of selective ion-responsive systems for pharmaceutical applications.

Ion-Sensitive Component	Incorporated Substance	Application	Ion-Induced Response	Ref.
MOFs	5-FU	Potential treatment of central nervous system diseases	Zn^2+^ dependent drug release	[46]
	5-FU	Potential treatment of central nervous system diseases	Zn^2+^ dependent drug release	[47]
	5-FU	n/a	Zn^2+^ and Ca^2+^ dependent drug release	[48]
Modified liposomal carriers	Dye	n/a	Ca^2+^ dependent drug release	[49]
	Chelating agent, fluorescein	Hg^2+^ neutralization and detection	Hg^2+^ dependent release	[50]
	Fluorescent dye	n/a	Cu^2+^ dependent release	[51]
D3F3 peptide	Doxorubicin	Possible prostate cancer treatment	Zn^2+^ dependent in situ hydrogel formation	[52]
Mesoporous silica nanoparticles modified with Pb^2+^-activated DNAzyme	Fluorescein	Pb^2+^ detection	Pb^2+^ dependent release	[53]
Prodrug 1,2,4-trioxolane moiety	ML4118S	Plasmodium infection treatment	Fe^2+^ dependent activation	[54]
Polynucleotide framework	AS1411 aptamer	Cancer treatment	K^+^ and pH dependent release on cellular membrane	[55]
Pectin	n/a	n/a	Ca^2+^ dependent gelling	[58]
Polyacrylamide hydrogels	n/a	adhesive materials	Ion-dependent adhesion	[59]
PNI-co-CF3-PT0.2-co-DDDEEKC0.2	n/a	Biodevices and artificial nanochannels	Ca^2+^ concentration dependent channels	[60]
Cholesteric liquid crystalline polymer	n/a	Fast calcium level test	Color change in presence of Ca^2+^	[56]
Single strain 30-nucleotide DNA absorbed on carbon nanotubes	n/a	Determination of Hg^2+^ concentration in biological systems	Hg^2+^ mediated shift in emission energy	[57]
(1,2-diaminocyclohexane) platinum (II)	Platinum derivatives	Antitumor activity	Cl^−^ induced intracellular activation of chemotherapeutic agent	[61]

## Data Availability

Not applicable.

## References

[1] Manzari M.T., Shamay Y., Kiguchi H., Rosen N., Scaltriti M., Heller D.A. (2021). Targeted drug delivery strategies for precision medicines. Nat. Rev. Mater..

[2] James H.P., John R., Alex A., Anoop K.R. (2014). Smart polymers for the controlled delivery of drugs—A concise overview. Acta Pharm. Sin. B.

[3] Kotze M.J., van Velden D.P., van Rensburg S.J., Erasmus R. (2009). Pathogenic mechanisms underlying iron deficiency and iron overload: New insights for clinical application. eJIFCC.

[4] Portbury S.D., Adlard P.A. (2017). Zinc signal in brain diseases. Int. J. Mol. Sci..

[5] Reid I.R., Gamble G.D., Bolland M.J. (2016). Circulating calcium concentrations, vascular disease and mortality: A systematic review. J. Intern. Med..

[6] Vardhan K.H., Kumar P.S., Panda R.C. (2019). A review on heavy metal pollution, toxicity and remedial measures: Current trends and future perspectives. J. Mol. Liq..

[7] Zia K.M., Tabasum S., Khan M.F., Akram N., Akhter N., Noreen A., Zuber M. (2018). Recent trends on gellan gum blends with natural and synthetic polymers: A review. Int. J. Biol. Macromol..

[8] Burke W. (2014). The ionic composition of nasal fluid and its function. Health (Irvine. Calif.).

[9] Fijorek K., Püsküllüoğlu M., Tomaszewska D., Tomaszewski R., Glinka A., Polak S. (2014). Serum potassium, sodium and calcium levels in healthy individuals—Literature review and data analysis. Folia Med. Cracov..

[10] Ruiz-Ederra J., Levin M.H., Verkman A.S. (2009). In situ fluorescence measurement of tear film [Na^+^], [K^+^], [Cl^−^], and pH in mice shows marked hypertonicity in aquaporin-5 deficiency. Investig. Ophthalmol. Vis. Sci..

[11] Cao S.L., Ren X.W., Zhang Q.Z., Chen E., Xu F., Chen J., Liu L.C., Jiang X.G. (2009). In situ gel based on gellan gum as new carrier for nasal administration of mometasone furoate. Int. J. Pharm..

[12] Belgamwar V.S., Chauk D.S., Mahajan H.S., Jain S.A., Gattani S.G., Surana S.J. (2009). Formulation and evaluation of in situ gelling system of dimenhydrinate for nasal administration. Pharm. Dev. Technol..

[13] Wang S., Chen P., Zhang L., Yang C., Zhai G. (2012). Formulation and evaluation of microemulsion-based in situ ion-sensitive gelling systems for intranasal administration of curcumin. J. Drug Target..

[14] Gänger S., Schindowski K. (2018). Tailoring formulations for intranasal nose-to-brain delivery: A review on architecture, physico-chemical characteristics and mucociliary clearance of the nasal olfactory mucosa. Pharmaceutics.

[15] Sun Y., Li L., Xie H., Wang Y., Gao S., Zhang L., Bo F., Yang S., Feng A. (2020). Primary studies on construction and evaluation of ion-sensitive in situ gel loaded with paeonol-solid lipid nanoparticles for intranasal drug delivery. Int. J. Nanomed..

[16] Hao J., Zhao J., Zhang S., Tong T., Zhuang Q., Jin K., Chen W., Tang H. (2016). Fabrication of an ionic-sensitive in situ gel loaded with resveratrol nanosuspensions intended for direct nose-to-brain delivery. Colloids Surfaces B Biointerfaces.

[17] Rajput A.P., Butani S.B. (2018). Fabrication of an ion-sensitive in situ gel loaded with nanostructured lipid carrier for nose to brain delivery of donepezil. Asian J. Pharm..

[18] Sun J., Zhou Z. (2018). A novel ocular delivery of brinzolamide based on gellan gum: In vitro and in vivo evaluation. Drug Des. Devel. Ther..

[19] Patel P., Patel G. (2021). Formulation, ex-vivo and preclinical in-vivo studies of combined ph and ion-sensitive ocular sustained in situ hydrogel of timolol maleate for the treatment of glaucoma. Biointerface Res. Appl. Chem..

[20] Yu S., Wang Q.M., Wang X., Liu D., Zhang W., Ye T., Yang X., Pan W. (2015). Liposome incorporated ion sensitive in situ gels for opthalmic delivery of timolol maleate. Int. J. Pharm..

[21] Sultana Y., Aqil M., Ali A. (2006). ion-activated, gelrite®-based in situ ophthalmic gels of pefloxacin mesylate: Comparison with conventional eye drops. Drug Deliv. J. Deliv. Target. Ther. Agents.

[22] Nayak N.S., Srinivasa U. (2017). Design and evaluation of ion activated in situ ophthalmic gel of moxifloxacin hydrochloride and ketorolac tromethamine combination using carboxy methylated tamarind kernel powder. Saudi J. Med. Pharm. Sci..

[23] Janga K.Y., Tatke A., Balguri S.P., Lamichanne S.P., Ibrahim M.M., Maria D.N., Jablonski M.M., Majumdar S. (2018). ion-sensitive in situ hydrogels of natamycin bilosomes for enhanced and prolonged ocular pharmacotherapy: In vitro permeability, cytotoxicity and in vivo evaluation. Artif. Cells Nanomedicine Biotechnol..

[24] Terreni E., Zucchetti E., Tampucci S., Burgalassi S., Monti D., Chetoni P. (2021). Combination of nanomicellar technology and in situ gelling polymer as ocular drug delivery system (Odds) for cyclosporine-a. Pharmaceutics.

[25] Göttel B., de Souza e Silva J.M., Santos de Oliveira C., Syrowatka F., Fiorentzis M., Viestenz A., Viestenz A., Mäder K. (2020). Electrospun nanofibers—A promising solid in-situ gelling alternative for ocular drug delivery. Eur. J. Pharm. Biopharm..

[26] Zhang X., Pan Y., Li S., Xing L., Du S., Yuan G., Li J., Zhou T., Xiong D., Tan H. (2020). Doubly crosslinked biodegradable hydrogels based on gellan gum and chitosan for drug delivery and wound dressing. Int. J. Biol. Macromol..

[27] Ng J.Y., Zhu X., Mukherjee D., Zhang C., Hong S., Kumar Y., Gokhale R., Ee P.L.R. (2021). Pristine gellan gum–collagen interpenetrating network hydrogels as mechanically enhanced anti-inflammatory biologic wound dressings for burn wound therapy. ACS Appl. Bio Mater..

[28] Bellini D., Cencetti C., Meraner J., Stoppoloni D., D’Abusco A.S., Matricardi P. (2015). An in situ gelling system for bone regeneration of osteochondral defects. Eur. Polym. J..

[29] Harish N., Prabhu P., Charyulu R., Gulzar M., Subrahmanyam E.V. (2009). Formulation and evaluation of *in situ* gels containing clotrimazole for oral candidiasis. Indian J. Pharm. Sci..

[30] Abd Ellah N.H., Abouelmagd S.A., Abbas A.M., Shaaban O.M., Hassanein K.M.A. (2018). Dual-responsive lidocaine in situ gel reduces pain of intrauterine device insertion. Int. J. Pharm..

[31] Hecht H., Srebnik S. (2016). Structural characterization of sodium alginate and calcium alginate. Biomacromolecules.

[32] Liu Z., Li J., Nie S., Liu H., Ding P., Pan W. (2006). Study of an alginate/HPMC-based in situ gelling ophthalmic delivery system for gatifloxacin. Int. J. Pharm..

[33] Pandya T.P., Modasiya M.K., Patel V.M. (2011). Sustained ophthalmic delivery of ofloxacin hydrochloride from an ion-activated in situ gelling system. Der Pharm. Lett..

[34] Shelley H., Rodriguez-Galarza R.M., Duran S.H., Abarca E.M., Babu R.J. (2018). In situ gel formulation for enhanced ocular delivery of nepafenac. J. Pharm. Sci..

[35] Kubo W., Miyazaki S., Attwood D. (2003). Oral sustained delivery of paracetamol from in situ-gelling gellan and sodium alginate formulations. Int. J. Pharm..

[36] Hu C., Feng H., Zhu C. (2012). Preparation and characterization of rifampicin-PLGA microspheres/sodium alginate in situ gel combination delivery system. Colloids Surfaces B Biointerfaces.

[37] Hori Y., Winans A.M., Irvine D.J. (2009). Modular injectable matrices based on alginate solution/microsphere mixtures that gel in situ and co-deliver immunomodulatory factors. Acta Biomater..

[38] Zhu Q., Wei Y., Li C., Mao S. (2018). Inner layer-embedded contact lenses for ion-triggered controlled drug delivery. Mater. Sci. Eng. C.

[39] Liu H., Zhu J., Bao P., Ding Y., Shen Y., Webster T.J., Xu Y. (2019). Construction and in vivo/in vitro evaluation of a nanoporous ion-responsive targeted drug delivery system for recombinant human interferon α-2b delivery. Int. J. Nanomedicine.

[40] Zhang B., Sun B., Li X., Yu Y., Tian Y., Xu X., Jin Z. (2015). Synthesis of pH- and ionic strength-responsive microgels and their interactions with lysozyme. Int. J. Biol. Macromol..

[41] Zheng Y., Sun J., Jin X., Wu X. (2018). Influence of ionic strength on the ph-sensitive in vitro ibuprofen release from dextran-poly(acrylic acid) copolymer. Indian J. Pharm. Sci..

[42] Bodmeier R., Guo X., Sarabia R.E., Skultety P.F. (1996). The influence of buffer species and strength on diltiazem HCl release from beads coated with the aqueous cationic polymer dispersions, eudragit RS, RL 30D. Pharm. Res..

[43] Dragan E.S., Cocarta A.I. (2016). Smart macroporous IPN hydrogels responsive to pH, temperature, and ionic strength: Synthesis, characterization, and evaluation of controlled release of drugs. ACS Appl. Mater. Interfaces.

[44] Jarvinen K., Akerman S., Svarfvar B., Tarvainen T., Viinikka P., Paronen P. (1998). Drug release from pH and ionic strength responsive poly(acrylic acid) grafted poly(vinylidenefluoride) membrane bags in vitro. Pharm. Res..

[45] An J., Geib S.J., Rosi N.L. (2009). Cation-triggered drug release from a porous zinc-adeninate metal-organic framework. J. Am. Chem. Soc..

[46] Tan L.L., Li H., Zhou Y., Zhang Y., Feng X., Wang B., Yang Y.W. (2015). Zn^2+^-triggered drug release from biocompatible zirconium MOFs equipped with supramolecular gates. Small.

[47] Du X., Fan R., Qiang L., Xing K., Ye H., Ran X., Song Y., Wang P., Yang Y. (2017). Controlled Zn^2+^-triggered drug release by preferred coordination of open active sites within functionalization indium metal organic frameworks. ACS Appl. Mater. Interfaces.

[48] Wu M.X., Gao J., Wang F., Yang J., Song N., Jin X., Mi P., Tian J., Luo J., Liang F. (2018). Multistimuli responsive core–shell nanoplatform constructed from Fe_3_O_4_@MOF equipped with pillar[6]arene nanovalves. Small.

[49] Lou J., Best M.D. (2020). Calcium-Responsive Liposomes: Toward ion-Mediated Targeted Drug Delivery.

[50] Yigit M.V., Mishra A., Tong R., Cheng J., Wong G.C.L., Lu Y. (2009). Inorganic mercury detection and controlled release of chelating agents from ion-responsive liposomes. Chem. Biol..

[51] Veremeeva P.N., Lapteva V.L., Palyulin V.A., Sybachin A.V., Yaroslavov A.A., Zefirov N.S. (2014). Bispidinone-based molecular switches for construction of stimulus-sensitive liposomal containers. Tetrahedron.

[52] Tao M., Liu J., He S., Xu K., Zhong W. (2019). In situ hydrogelation of forky peptides in prostate tissue for drug delivery. Soft Matter.

[53] Song W., Li J., Li Q., Ding W., Yang X. (2015). Avidin-biotin capped mesoporous silica nanoparticles as an ion-responsive release system to determine lead(II). Anal. Biochem..

[54] Deu E., Chen I.T., Lauterwasser E.M.W., Valderramos J., Li H., Edgington L.E., Renslo A.R., Bogyo M. (2013). Ferrous iron-dependent drug delivery enables controlled and selective release of therapeutic agents in vivo. Proc. Natl. Acad. Sci. USA.

[55] Peng P., Wang Q., Du Y., Wang H., Shi L., Li T. (2020). Extracellular ion-responsive logic sensors utilizing DNA dimeric nanoassemblies on cell surface and application to boosting AS1411 internalization. Anal. Chem..

[56] Moirangthem M., Arts R., Merkx M., Schenning A.P.H.J. (2016). An optical sensor based on a photonic polymer film to detect calcium in serum. Adv. Funct. Mater..

[57] Heller D.H., Jeng E.S., Yeung T.-K., Martinez B.M., Moll A.E., Gastala J.B., Strano M.S. (2006). Optical detection of DNA conformational polymorphism on single-walled carbon nanotubes. Science.

[58] Castile J., Cheng Y.H., Simmons B., Perelman M., Smith A., Watts P. (2013). Development of in vitro models to demonstrate the ability of PecSys®, an in situ nasal gelling technology, to reduce nasal run-off and drip. Drug Dev. Ind. Pharm..

[59] Nakamura T., Takashima Y., Hashidzume A., Yamaguchi H., Harada A. (2014). A metal-ion-responsive adhesive material via switching of molecular recognition properties. Nat. Commun..

[60] Li Y., Xiong Y., Wang D., Li X., Chen Z., Wang C., Qin H., Liu J., Chang B., Qing G. (2019). Smart polymer-based calcium-ion self-regulated nanochannels by mimicking the biological Ca^2+^-induced Ca^2+^ release process. NPG Asia Mater..

[61] Murakami M., Cabral H., Matsumoto Y., Wu S., Kano M.R., Yamori T., Nishiyama N., Kataoka K. (2011). Improving drug potency and efficacy by nanocarrier-mediated subcellular targeting. Sci. Transl. Med..

[62] Li J., Kataoka K. (2021). Chemo-physical Strategies to Advance the in vivo functionality of targeted nanomedicine: The next generation. J. Am. Chem. Soc..

[63] Smith G.L., Eisner D.A. (2019). Calcium buffering in the heart in health and disease. Circulation.

[64] Litan A., Langhans S.A. (2015). Cancer as a channelopathy: Ion channels and pumps in tumor development and progression. Front. Cell. Neurosci..

[65] Bagur R., Hajnóczky G. (2017). Intracellular Ca^2+^ sensing: Role in calcium homeostasis and signaling. Mol. Cell.

[66] Bafaro E., Liu Y., Xu Y., Dempski R.E. (2017). The emerging role of zinc transporters in cellular homeostasis and cancer. Signal Transduct. Target. Ther..

